# Supplementary material for: Interrelationship Between Cardiopulmonary Exercise Testing Indices and Markers of Subclinical Cardiovascular Dysfunction in Those with Type 2 Diabetes—An Observational Cross-Sectional Analysis
Source: J Funct Morphol Kinesiol. 2025 Sep 26;10(4):371. doi: 10.3390/jfmk10040371 (PMC12551124; doi:10.3390/jfmk10040371)
Supplement: Supplementary file 1 [file jfmk-10-00371-s001.zip › jfmk-3794644-supplementary.pdf]

Association of Novel Indices of Cardiopulmonary Exercise Testing with Measures of Cardiovascular Remodelling in Asymptomatic Type 2 Diabetes: Supplementary Material

Table S1. Exploratory analysis with BMI as a covariate and the analysis also run on healthy matched controls

	E/e'		LV mass: volume (g/ml)		Mean Aortic Distensibility (mmHg^{-1}x10^{3})		Peak Early Diastolic Strain Rate (S^{-1})		Myocardial Perfusion Reserve	
	**T2D**	**HV**	**T2D**	**HV**	**T2D**	**HV**	**T2D**	**HV**	**T2D**	**HV**
	SlopeV$_E$/VCO$_2$									
**β**	0.023	0.026	0.001	-0.001	0.041	0.219	0.002	0.012	0.001	-0.011
**95% CI**	[-0.087, 0.133]	[-0.103, 0.156]	[-0.005, 0.007]	[-0.011, 0.009]	[0.039, 0.121]	[0.048, 0.389]	[-0.006, 0.009]	[-0.002, 0.026]	[-0.043, 0.046	[-0.108, 0.07]
P	*0.681*	*0.682*	*0.738*	*0.808*	*0.315*	*0.052*	*0.667*	*0.081*	*0.949*	*0.663*
**ES**	0.003	0.006	0.001	0.002	0.013	0.192	0.002	0.098	<0.0001	0.007
	VO$_2$ Recovery (%)									
**β**	-0.104	-0.049	-0.05	0.005	0.218	0.054	-0.081	-0.001	-0.112	-0.01
**95% CI**	[0.117, 0.050]	[-0.131, 0.033]	[-0.006, 0.004]	[-0.001, 0.001]	[0.000, 0.132]	[-0.068, 0.177]	[-0.009, 0.004]	[-0.010, 0.009]	[-0.054, 0.021]	[-0.067, 0.046]
P	*0.43*	*0.232*	*0.695*	*0.114*	*0.049*	*0.371*	*0.481*	*0.845*	*0.388*	*0.707*
**ES**	0.009	0.046	0.002	0.079	0.05	0.027	0.006	0.001	0.011	0.005
	HR Recovery (%)									
**β**	-0.146	0.076	-0.007	0.001	0.096	0.117	0.0002	0.005	0.033	-0.0002
**95% CI**	[-0.275, -0.017]	[-0.005, 0.158]	[-0.013, -0.0004]	[-0.005, 0.008]	[0.004, 0.189]	[-0.002, 0.235]	[-0.009, 0.009]	[-0.005, 0.014]	[-0.02, 0.086]	[-0.062, 0.058]
P	*0.027*	*0.066*	*0.037*	*0.739*	*0.042*	*0.053*	*0.96*	*0.33*	*0.214*	*0.955*
**ES**	0.071	0.108	0.054	0.004	0.052	0.123	<0.0001	0.032	0.023	0.0001
	HR Reserve (%)									
**β**	0.007	0.0001	0.000005	0.0005	0.005	0.023	0.0003	-0.0005	0.007	-0.002
**95% CI**	[-0.012, 0.025	[-0.013, 0.014]	[-0.001, 0.001]	[-0.001, 0.001]	[-0.008, 0.019]	[0.005, 0.041]	[0.001, 0.002]	[-0.002, 0.001]	[-0.001, 0.015]	[-0.011, 0.007]
P	*0.48*	*0.987*	*0.99*	*0.357*	*0.454*	*0.051*	*0.587*	*0.545*	*0.071*	*0.665*
**ES**	0.007	<0.0001	<0.0001	0.028	0.007	0.199	0.004	0.012	0.048	0.007
	VO$_2$VT (ml/kg/min)									
**β**	-0.031	-0.005	-0.002	-0.003	-0.111	-0.055	-0.125	-0.002	0.273	-0.021
**95% CI**	[-0.244, 0.189]	[0.012, 0.007]	[-0.011, 0.011	[0.012, 0.007	[-0.244, 0.072]	[-0.244, 0.134]	[-0.024, 0.006]	[-0.016, 0.012]	[0.017, 0.183]	[-0.111, 0.068]
P	*0.801*	*0.575*	*0.988*	*0.575*	*0.281*	*0.557*	*0.236*	*0.799*	*0.018*	*0.629*
**ES**	0.001	0.01	<0.0001	0.01	0.015	0.012	0.018	0.002	0.08	0.008
	COP									
**β**	0.016	0.065	-0.002	-0.008	0.117	0.181	0.012	0.004	-0.042	0.002
**95% CI**	[0.179, 0.211]	[-0.091, 0.222]	[-0-011, 0.008]	[-0.02, 0.004]	[-0.021, 0.255]	[-0.041, 0.404]	[-0.002, 0.025]	[-0.014, 0.022]	[-0.121, 0.036]	[-0.108, 0.112]
P	*0.868*	*0.401*	*0.728*	*0.172*	*0.095*	*0.107*	*0.082*	*0.675*	*0.286*	*0.968*
**ES**	0.0004	0.024	0.002	0.061	0.035	0.087	0.018	0.006	0.017	<0.0001

Associations Between Novel CPET Variables and Cardiovascular Structure and Function Data is derived from generalised linear modelling where each cardiovascular variable was selected as a dependent variable. Data are as β, 95% confidence interval (CI) of β, P-value. Abbreviations: ES: Effect size (Partial Eta Squared) VE: minute ventilation; V_{CO2}: Carbon Dioxide Output; V_E/V_{CO2}: ventilatory efficiency; $\dot{V}O_2$: oxygen uptake; HR: heart rate; VT: ventilatory threshold; COP: cardiorespiratory optimal point; E/e': Mitral inflow velocity/ mitral annular early diastolic velocity; LV: left ventricular; AOD; aortic distensibility; PEDSR: peak early diastolic strain rate; MPR: myocardial perfusion reserve.

Table S2. Medication usage of those with type-2 diabetes and obesity

Medication	N
Angiotensin-Converting Enzyme Inhibitors	27
Alpha-Blockers	3
Angiotensin-II Receptor Blockers	11
Beta Blockers	6
Calcium Channel Blockers	19
Statins	55
Fibrates	3
Metformin	76
Sulphonylurea	12
Glucagon-like Peptide-1 Receptor Agonists	8
Gliptin	16
Sodium-Glucose Co-Transporter-2 Inhibitors	10
Insulin	0
